# Prospective policy analysis of healthcare workforce policies for effective non-communicable disease prevention and management in Meghalaya, India

**DOI:** 10.1093/heapol/czaf107

**Published:** 2026-06-29

**Authors:** Ankur Nair, Pratheeba John, Rajeev Sadanandan

**Affiliations:** Health Systems Transformation Platform-India, AISF Building, First Floor, Kalka Devi Marg, Lajpat Nagar IV, New Delhi 110024, India; Health Systems Transformation Platform-India, AISF Building, First Floor, Kalka Devi Marg, Lajpat Nagar IV, New Delhi 110024, India; Health Systems Transformation Platform-India, AISF Building, First Floor, Kalka Devi Marg, Lajpat Nagar IV, New Delhi 110024, India

**Keywords:** human resources for health, health policy, non-communicable diseases, health equity, social justice, India, Meghalaya, prospective policy analysis, scenario planning, health governance

## Abstract

This study presents a prospective policy analysis of healthcare workforce (human resources for health, HRH) reforms in Meghalaya, India, a state facing dual challenges of a rising burden of non-communicable diseases (NCDs) and persistent inequities in access to medical and public health specialists. Grounded in a health systems and social justice perspective, this study examines current HRH needs and proposed reforms, evaluating their potential to address the evolving health demands of the population, particularly among poor and marginalized groups vulnerable to NCDs. Using secondary evidence, including national surveys, epidemiological data, and government reports, the study maps workforce gaps, especially in rural and tribal regions, and highlights the physician-centric nature of HRH governance in India. The Walt and Gilson policy triangle, combined with adapted HRH evaluation frameworks, guided the analysis of policy context, content, processes, and actors. This approach identified key gaps in recruitment, rural retention, cadre structures, and specialist training for effective NCD prevention and management. A scenario analysis, using the Intuitive Logic method, developed a best-case trajectory of reform achievable through equity-oriented design, strong political will, and institutional capacity, while recognizing risks of policy stagnation. Findings indicate that achieving a socially accountable HRH system characterized by equitable distribution, transparent governance, and meaningful community engagement is critical for improving NCD outcomes and reducing health inequities, particularly by preventing financial distress among the poor. The study contributes to health policy and systems research by linking institutional design to future health system resilience, underscoring the need to embed justice, participation, and adaptability within HRH governance to effectively address present and future health challenges in low-resource contexts such as Meghalaya.

Key messages

- Human resource constraints, particularly limited specialist availability and inequitable rural deployment, remain a major barrier to managing the rising burden of non-communicable diseases (NCDs) and addressing health inequities in Meghalaya, India.
- Tackling these challenges requires more than administrative reforms. Meghalaya's proposed human resources for health (HRH) policy introduces elements like dedicated Public Health and Specialist cadres, crucial for NCD prevention and management, but its transformative potential depends on addressing past policy gaps through inclusive governance, local adaptation, and robust implementation.
- Achieving an equity-focused HRH system that can mitigate the impact of NCDs on vulnerable populations requires sustained political will, adequate funding, and an explicit equity orientation. Policy design alone is insufficient to overcome existing workforce gaps, especially in tribal and remote communities.
- Meghalaya’s experience offers broader lessons for health systems: transparent recruitment, rural-focused incentives, community-responsive training, and dedicated cadres for public health and specialist NCD care are vital for building more equitable health workforces, especially in low-resource contexts facing complex health transitions.

## Introduction

The global surge in non-communicable diseases (NCDs) presents a formidable challenge to health systems, particularly in low- and middle-income countries (LMICs), where limited resources and demographic shifts strain service delivery (WHO 2023). Effective NCD prevention and management require a skilled and equitably distributed health workforce (Frenk et al. 2010). However, human resources for health (HRH) crises characterized by shortages, maldistribution, and inadequate skill mix are widespread across LMICs, undermining universal health coverage and compounding the NCD burden (Anyangwe and Mtonga 2007, Crisp and Chen 2014, WHO 2016).

In India, health systems have historically been physician-oriented, with recruitment and distribution shaping access to care (Reddy et al. 2011, Rao et al. 2012). HRH deficits disproportionately affect rural and disadvantaged populations, exacerbating inequities (McIntyre and Kutzin 2016). Once considered ‘diseases of affluence’, NCDs now disproportionately affect the poor in LMICs (Reddy 2002, Marmot 2005, Bukhman et al. 2020). Conventional risk-factor models inadequately explain these trends; structural determinants such as poverty, isolation, and inequitable access are central (Niessen et al. 2018). Vulnerable groups often lack access to preventive services and timely care, leading to severe complications and financial hardships, reinforcing poverty cycles (Kankeu et al. 2013). HRH reforms alone cannot ensure equity; specialist expansion requires infrastructure, financing, and attention to social determinants.

India faces critical HRH shortages, with a workforce density of 20.6 per 10 000 well below the World Health Organization (WHO) threshold of 44.5 (WHO 2016, Karan et al. 2021). Stark interstate and rural-urban disparities further constrain access (Rao et al. 2012, Anand and Fan 2016). Meghalaya, a geographically complex, predominantly rural, tribal-majority state, exemplifies these challenges. The state has undergone a marked epidemiological transition, with non-communicable diseases increasingly dominating the disease burden since 1990 (ICHMR, PHFI, & IHME, 2017) . Cardiovascular diseases and cancers are major drivers of mortality, with East Khasi Hills recording some of India’s highest tobacco-related cancer rates (Mathur et al. 2020). The state also faces concurrent substance use and HIV challenges (Ambekar et al. 2019), adding complexity to workforce planning. These burdens intersect with poverty, isolation, and gendered access, constraining equitable use of services. Since NCDs require sustained and coordinated management, HRH reforms must align with broader shifts from episodic to integrated chronic care models within health systems.

These pressures are compounded by severe HRH deficits. Meghalaya has fewer than 11 skilled health workers per 10 000 population, with even lower densities in rural areas (IQVIA and MHSSP 2022a). While recruitment has been decentralized and new cadres created, challenges in equitable deployment and retention persist, particularly in remote districts, reflecting structural governance constraints that entrench inequities.

Against this backdrop, the Government of Meghalaya has proposed strategic HRH reforms. These include establishing a public health cadre to strengthen preventive and community health functions, restructuring clinical cadres to address specialist gaps, rationalizing postings and rural incentives, improving transfer and posting policies for transparency and stability, and building HRH information systems for planning and monitoring. These reforms build on a longer trajectory of state policymaking, including the Meghalaya Health Service Rules (1982, 1990), the Meghalaya Health Policy (2021), and recent cadre restructuring and posting policies.

This study undertakes prospective policy analysis of these proposed HRH reforms to assess their potential to address workforce gaps and strengthen NCD care from an equity perspective. Using a scenario-building approach informed by policy document analysis and informal consultations, it identifies actionable strategies both within and beyond current commitments. In doing so, the study contributes to global debates on designing HRH reforms in LMICs to tackle rising NCD burdens alongside entrenched inequities.

## Methods

### Study design

This study employed a prospective policy analysis approach, defined as a forward-looking assessment that anticipates challenges, explores alternative futures, and informs decision-making before implementation (Buse 2008, Paina and Peters 2012). The design was selected to examine Meghalaya’s evolving HRH policy landscape in the context of its rising NCD burden. Unlike retrospective assessments, this approach was adopted to anticipate systemic challenges, particularly those shaped by shifting epidemiological trends and HRH disruptions following the COVID-19 pandemic.

Health policy analysis in LMICs has historically drawn on diverse methodological traditions, yet coherence and transparency in design remain as challenges (Gilson and Raphaely 2008). To address this, the study adopted a consolidated two-tier design: Walt and Gilson’s Policy Triangle was applied for retrospective policy mapping, and the Intuitive Logic method was used to construct forward-looking scenarios.

The study was conducted between May 2023 and July 2024, during the drafting of Meghalaya’s HRH reforms, with the aim of identifying risks and opportunities before finalization. Its purpose was to construct a normative best-case scenario, highlighting governance, equity, and system requirements for effective implementation. In doing so, the analysis generated actionable insights to strengthen adaptive planning and ensure that workforce reforms contribute not only to filling HRH gaps but also to advancing equitable service delivery.

### Data sources

This study drew on a diverse set of materials to triangulate insights and ensure comprehensive coverage of the HRH and NCD policy landscape in Meghalaya. Document searches and retrieval were conducted between May and December 2023, covering sources from 1982 to 2023. No primary data were collected for this study. A limited number of informal consultations were undertaken solely to validate analytical frameworks and scenario assumptions; these did not involve systematic data collection or analysis. Key sources included policy documents, government and technical reports, peer-reviewed literature, and grey literature (Table 1).

**Table 1:** Data sources informing the policy and scenario analysis.

Category	Description	Sources
Policy and institutional documents	Official documents outlining HRH policy frameworks, governance structures, recruitment and deployment regulations, bond obligations, and draft policy revisions	Websites of NHM Meghalaya; MoHFW, Government of India; Department of Health and Family Welfare, Government of Meghalaya
Health systems and workforce data	Statistical data on workforce availability, distribution, infrastructure, and institutional assessments of HRH management systems	Rural Health Statistics (RHS); WHO Global Strategy on Human Resources for Health: Workforce 2030 (2016); National Health Systems Resource Centre (NHSRC) reports; Meghalaya Health Systems Strengthening Project (MHSSP) surveys and reports
Disease burden and risk-factor data	Epidemiological evidence on NCD burden, behavioural risk factors, and district-level health outcomes	GBD 2019; National Family Health Survey-5 (2019–20); district health bulletins; cancer registries; NCD cell reports; NGO surveillance data

The study draws exclusively on secondary data sources listed above, supplemented by informal consultations used for validation. No new primary data were collected.

NHM, National Health Mission; MoHFW, Ministry of Health and Family Welfare; RHS, Rural Health Statistics; NHSRC, National Health Systems Resource Centre; MHSSP, Meghalaya Health Systems Strengthening Project; HRH, Human Resources for Health; GBD, Global Burden of Disease; NCD, non-communicable disease.

### Stakeholder consultations

To ensure contextual relevance, informal consultations were held at three points (initial, mid-phase, and final) with state health officials, HRH planners, and district-level health workers. These sessions aimed to clarify the scope and intent of the HRH reforms, validate analytical variables and scenario assumptions, and strengthen policy relevance and contextual grounding. Consultations were brief purposive sessions (30–60 min), conducted in person or virtually with individuals or small groups. They were not audio recorded; instead, contemporaneous non-attributable memos were prepared. Verbal consent was obtained after explaining the purpose, voluntary participation, and limited use of inputs (framework validation rather than systematic data collection). No identifying or sensitive information was recorded, and where feasible, summary notes were member checked for accuracy.

### Analytical steps

**Figure 1:**
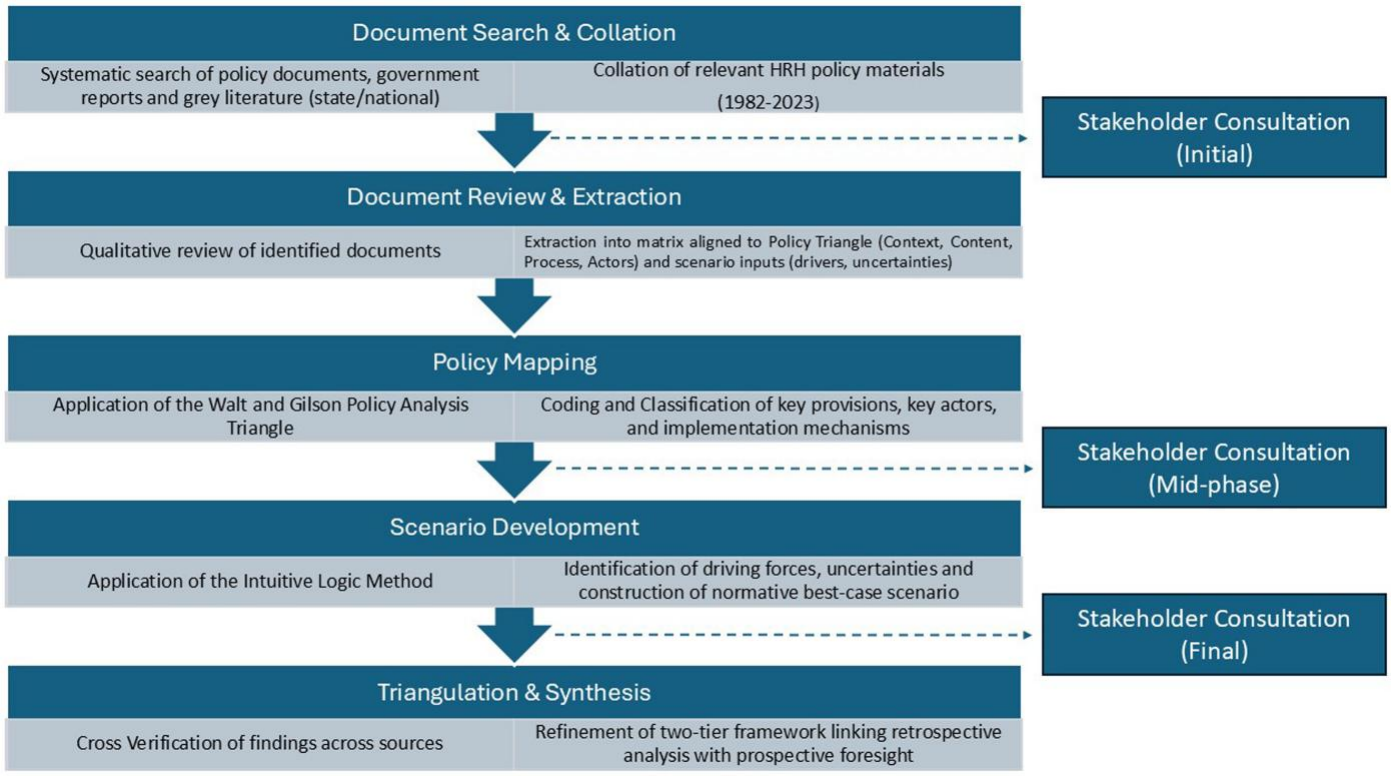
Analytical process flowchart for the study. This flowchart illustrates the five-stage process of the prospective policy analysis: document review and extraction, policy mapping, scenario development, triangulation, and consultation validation. Source: Authors’ own illustration.

The analysis followed a structured process integrating retrospective policy mapping with prospective scenario development. Following document search and collation, the first analytical stage was document review and extraction, where identified materials were qualitatively assessed and key variables extracted into a matrix aligned with the Walt and Gilson Policy Analysis Triangle (Context, Content, Process, Actors) and scenario-building inputs (drivers, assumptions, uncertainties). To ensure rigour, two researchers reviewed documents independently and resolved discrepancies through discussion (Fig. 1).

In the second stage, policy mapping involved coding and classifying materials to identify policy provisions, actor roles, and implementation mechanisms, guided by the Policy Analysis Triangle. The third stage applied the Intuitive Logic method to identify driving forces and uncertainties and to construct a normative best-case scenario specifying enabling conditions for equity-oriented implementation, including governance, financing, institutional capacity, and stakeholder engagement. The fourth stage, triangulation and synthesis, involved cross-verification of findings across documents, reports, and literature, consolidating outputs into a two-tier framework linking retrospective analysis with prospective foresight. Throughout the process, stakeholder consultations informed the analysis at three points: initial (to clarify scope), mid-phase (to validate variables and assumptions), and final (to sense-check the scenario).

### Conceptual and analytical framework

The study was informed by a conceptual understanding of health workforce dynamics situated within broader social, economic, and political contexts, drawing on the system-oriented framework proposed by Sonderegger et al. (2021).

**Figure 2:**
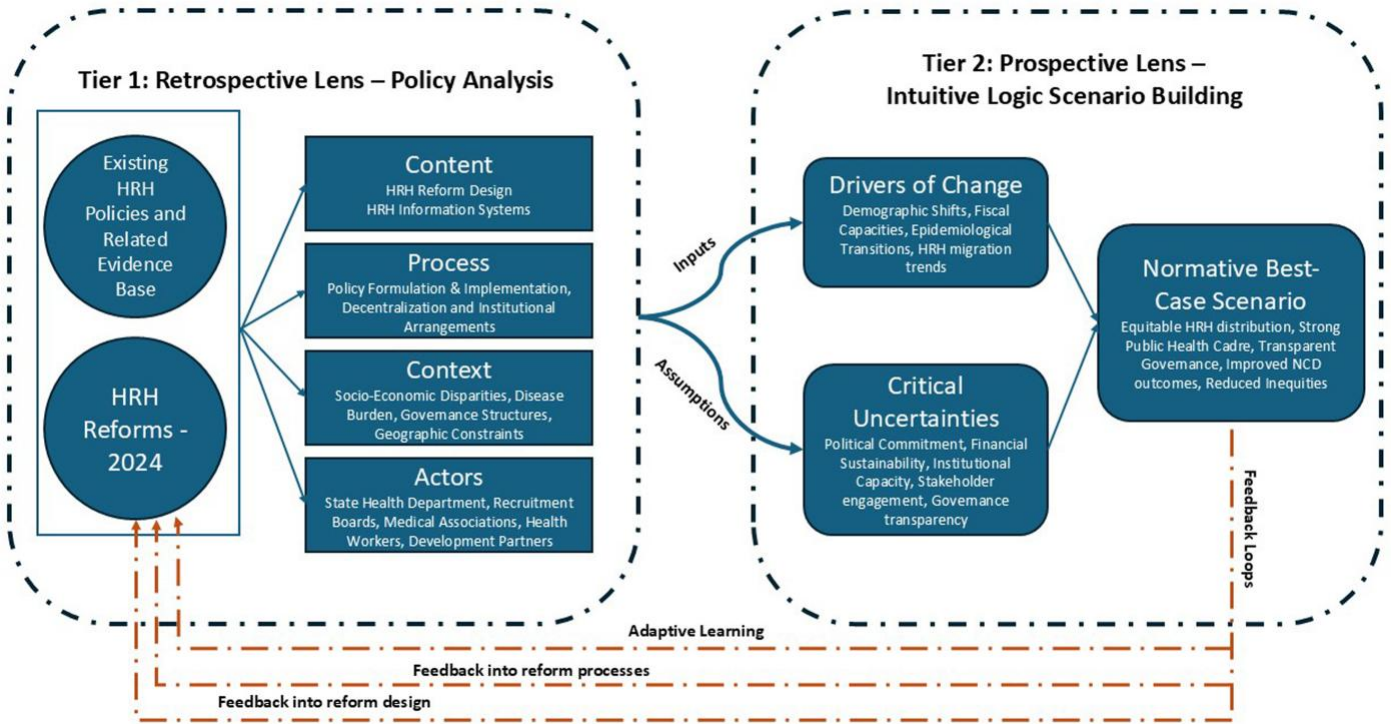
Two-tier analytical framework for this study. This framework combines Walt and Gilson’s Policy Analysis Triangle for retrospective policy mapping (Tier 1) with Intuitive Logic scenario building for prospective foresight (Tier 2), supported by equity and systems thinking lenses.Source:Authors’ own illustration.

For the core analysis, a two-tier framework integrated retrospective policy mapping with prospective scenario building. Tier 1 drew on the Policy Analysis Triangle (1994) to examine the context, content, process, and actors shaping HRH reform design and implementation in Meghalaya. Tier 2 employed the Intuitive Logic approach (Buse 2008, Paina and Peters 2012) to identify key drivers of change and uncertainties, and to construct a normative best-case scenario for equitable and effective reform (Fig. 2).

This combined framework served two purposes. First, it enabled a backward-looking assessment of institutional arrangements, policy content, and actor dynamics that have historically shaped HRH reform in Meghalaya. Second, it provided a forward-looking exploration of the enabling conditions required for successful and equity-oriented implementation of proposed reforms. Informal stakeholder consultations at three stages (initial, mid-phase, and final) further refined assumptions, validated scenario inputs, and ensured contextual relevance. By linking retrospective insights with prospective foresight, the framework illustrates how scenario-based approaches can complement traditional policy analysis in low-resource, politically complex settings, thereby contributing to the broader health policy and systems research (HPSR) agenda on adaptive, equity-oriented governance in LMICs.

### Scenario building

A central component of this analysis was the application of scenario building using the Intuitive Logic method to explore plausible futures for HRH development in Meghalaya. This method was selected because it provides a structured way to identify driving forces, uncertainties, and alternative futures, making it particularly useful in policy contexts of uncertainty (Buse 2008, Paina and Peters 2012).

The Intuitive Logic method was applied not to forecast outcomes but to construct a best-case scenario, an aspirational roadmap illustrating the potential of the Proposed 2024 HRH Reforms if implemented with strong fidelity to equity principles. While moderate and worst-case outcomes were acknowledged, the analysis deliberately concentrated on delineating enabling conditions for achieving an equitable and effective HRH system.

The best-case scenario drew on trends and constraints identified in the document review (e.g. workforce availability, disease burden, and infrastructure); enabling conditions outlined in the Proposed HRH Policy, such as political commitment, financing, institutional capacity, stakeholder engagement, and transparent governance; and validation from stakeholder consultations confirming the plausibility and contextual relevance of assumptions.

The resulting scenario projects how successful implementation of the reforms, coupled with explicit equity integration, could strengthen HRH capacity, improve the distribution of health workers (particularly specialists in underserved areas), enhance health system responsiveness to NCDs, and advance health equity. The structured outputs of this scenario-building exercise are presented in Table 2, which outlines key assumptions, enabling conditions, and projected HRH and equity-related outcomes.

**Table 2:** Projected enablers, HRH outcomes, and equity impacts under the best-case scenario of Meghalaya’s 2024 HRH reforms.

Component	Scenario assumptions/design features	HRH system outcomes (projected)	Equity and NCD impact (projected)
**Foundational enablers**			
Political will and leadership	Sustained political commitment; leadership actively resources and champions reforms	Reforms prioritized and insulated from interference; institutionalization of policy processes	Creates enabling environment for equity-oriented HRH and NCD system responsiveness
Resource allocation and fiscal space	Adequate, equitably allocated, and consistently available funding (cadres, rural incentives, NCD programmes, infrastructure)	Competitive remuneration; full operationalization of reforms; stable support for rural service	Improved availability of NCD services across districts; reduced financial barriers for disadvantaged groups
Institutional and implementation capacity	Strengthened capacity of Department of Health and Family Welfare, Medical Recruitment Board, and State Transfer Committee	Transparent recruitment; rational deployment; timely grievance redressal; robust HRH information systems	Strengthened governance for equitable NCD service delivery
Stakeholder engagement and multi-sectoral collaboration	Continuous engagement of health workers, associations, CSOs, and communities; coordination with education, transport, rural development	Socially accountable and context-relevant HRH policies; inter-sectoral support for HRH retention	Community-owned, culturally appropriate NCD prevention and care; structural determinants more effectively addressed
Transparency and governance	Clear rules for recruitment, transfers, promotions, and resource allocation; strong monitoring and evaluation with disaggregated data	Increased trust in HRH management; evidence-informed adjustments to policy and practice	Enhanced public confidence in health system, measurable progress towards equity
**Implementation of HRH reforms**			
Functional sub-cadres (Specialist, Public Health, Teaching)	Cadres fully operational with defined roles, leadership, and career pathways; mandatory CME and in-service training	Optimized skill use, improved motivation, expanded NCD-specific expertise, sustainable in-state HRH production	Expanded scope and quality of NCD services; greater emphasis on prevention and health promotion
Effective transfer and posting policy	Facility categorization (accessible, difficult, inaccessible) applied; CRP linked to finalized incentives; transparent EHRMS-driven transfers	Equitable distribution of doctors and specialists; reduced vacancies in peripheral areas; improved retention	Equitable NCD access across districts; narrowing of geographic disparities in outcomes
Optimized HRH production and entry	Increased in-state production of doctors and specialists; streamlined recruitment via Medical Recruitment Board; selective *ad hoc*/lateral entry for high-need specialities	Reduced shortages; alignment of skills with population health needs	Sustainable workforce pipeline for NCDs and priority health conditions
Enhanced workforce competencies and support	Mandatory CME and NCD-focused in-service training; task-shifting for screening and management; improved infrastructure and working conditions	Workforce equipped for NCD prevention and culturally competent care; strengthened role of nurses and CHWs; higher morale	Early detection and improved management of NCDs at primary level; reduced reliance on tertiary specialist care

Outlines scenario assumptions, projected HRH outcomes, and their equity and NCD care implications.

CME, continuing medical education; CSOs, civil society organizations; CRP, compulsory rural posting; CHW, community health worker; HRH, human resources for health; EHRMS, electronic human resource management system; NCD, non-communicable diseases.

### Ethical considerations

This study primarily analysed publicly available documents, reports, and policy papers and did not involve systematic primary data collection with human participants. A limited number of informal consultations were undertaken solely to validate the analytical framework and scenario assumptions. For these consultations, verbal consent was obtained after explaining the study purpose, voluntary participation, and intended use of inputs. No sensitive or personally identifying information was collected; anonymity and confidentiality were maintained, and summary notes were member-checked for accuracy.

In line with institutional and international guidelines for HPSR, formal ethics approval was not required, as the study involved minimal risk and no identifiable personal data. The research adhered to principles of responsible conduct, transparency, and respect for participants’ perspectives.

### Positionality statement

The research team conducted this study in their capacity as technical partners to the Government of Meghalaya during the period of HRH policy reform. This role provided valuable contextual understanding and access to relevant documents but also required a reflexive stance to guard against assumptions of insider knowledge or normative bias.

Members of the team have prior experience engaging with health systems and policy processes in India, including the North-East region. Informal consultations with state-level stakeholders were therefore used not as data collection but as a means of grounding the analytical framework and testing assumptions.

Throughout the study, efforts were made to maintain transparency in analytical choices, acknowledge contextual constraints, and ensure that interpretations remained anchored in documented evidence. This reflexive approach is consistent with guidance in HPSR, which emphasizes the importance of positionality, reflexivity, and contextual sensitivity in policy analysis (Gilson 2012).

## Results

This prospective policy analysis provides a structured examination of Meghalaya’s Proposed 2024 HRH Reforms, with attention to their potential implications on health equity and the management of NCDs. The analysis is organized into four interrelated domains, namely contextual landscape, reform content, processes and actors, and a prospective scenario analysis on NCD care and equity, which reflect key dimensions of HPSR.

### Contextual landscape: human resources for health challenges, non-communicable disease burden, and inequities in Meghalaya—a systems and equity lens

Meghalaya’s HRH policy architecture has evolved incrementally over the past four decades. The 1982 and 1990 Service Rules established recruitment foundations, while the 2021 Health Policy and 2023 cadre restructuring marked recognition of HRH as central to system performance (Government of Meghalaya 1982, 1990, 2021, 2023a, 2023b). The Proposed 2024 HRH Reforms thus emerge as the most comprehensive attempt to restructure workforce governance in the state, with explicit implications for NCD management and health equity. Crucially, the trajectory of HRH policy reform in Meghalaya intersects with entrenched structural inequities and shifting disease burdens, underscoring the need to situate these reforms within the state’s broader demographic and epidemiological realities.

Meghalaya’s HRH challenges are best understood as socially embedded and politically mediated, consistent with health systems thinking and social determinants frameworks (CSDH 2008, de Savigny and Adam 2009, Bukhman et al. 2020). Quantitative insights from recent HRH enumeration and specialist assessments (IQVIA and MHSSP 2022a) further contextualize these dynamics.

Meghalaya’s HRH challenges are deeply shaped by its structural, geographic, and socio-political context. Around 80% of the state’s population lives in rural areas, and its rugged terrain combined with a predominantly matrilineal tribal social structure (Khasis, Garos, and Jaintias) contributes to spatial fragmentation in healthcare access (Census of India 2011). High poverty rates, limited educational opportunities, and weak institutional infrastructure further constrain both health-seeking behaviour and the production of locally trained health workers.

The HRH Enumeration Report (IQVIA and MHSSP 2022a) highlights persistent infrastructural deficits: over half of facilities face gaps in reliable power, water, or internet, with one in five lacking electricity altogether (IQVIA and MHSSP 2022a). These gaps disproportionately affect peripheral health and wellness centres and complicate continuity of care for NCDs, which require sustained diagnostics, follow-up, and treatment.

Epidemiological transition has intensified these challenges. NCDs now account for 56% of total disability-adjusted life years (DALYs) in Meghalaya, up from 28% in 1990 (IHME 2022). Risk behaviours such as alcohol, tobacco, and betel nut use remain widespread (NFHS-5 2021), yet structural determinants—poverty, marginalization, and inequitable access—are more decisive drivers (Bukhman et al. 2020). This shift exposes a systemic misalignment. Meghalaya’s workforce remains trained and oriented towards acute care rather than chronic disease management, which requires long-term, team-based, integrated care models (WHO 2002). The shortage of specialists highlights limited adaptive capacity in the face of these demographic and epidemiological shifts (Dussault and Franceschini 2006).

Workforce shortages and inequities compound these systemic gaps. Meghalaya has 23.8 allopathic doctors per 100 000 population, well below the national average of 61.5 (RHS 2021). Fewer than half of 320 sanctioned specialist posts are filled, with most concentrated in East Khasi Hills, leaving some districts without a single specialist (IQVIA and MHSSP 2022c). This horizontal inequity, where populations with similar health needs receive differential care, reflects weak deployment governance and the lack of effective rural incentives (O'Donnell et al. 2008). Importantly, specialist availability alone does not guarantee equity; without functional facilities, referral linkages, and supportive social determinants, access for marginalized groups may remain constrained. Gender disparities further shape HRH distribution; women comprise most of the workforce but only 5% of specialists, reflecting barriers to advancement (George 2007, IQVIA and MHSSP, 2022a, 2022b, 2022c).

Finally, Meghalaya’s pluralistic health system, including public, private, faith-based, and traditional providers, faces institutional fragmentation. Despite formal commitments such as allocating 4% of GSDP to health (Meghalaya Health Policy, 2021), weak inter-sectoral coordination and governance capacity have constrained systemic improvements (Bennett et al. 2018). For the Proposed 2024 HRH Reforms to avoid technocratic limitations, they must integrate these structural determinants, strengthen inter-sectoral coordination, and foreground equity in both policy content and implementation (Sheikh et al. 2014).

### Evaluating human resources for health reforms through a social justice and systems lens

The evolution of Meghalaya’s HRH policy framework reveals both persistent systemic constraints and emerging opportunities for transformation. Historically governed by the outdated Meghalaya Health Services Rules (1982/1990), the system lacked differentiated cadres, equitable deployment mechanisms, and modern training and promotion pathways. This legacy has undermined responsiveness to contemporary public health challenges, particularly the rising burden of NCDs, which require both specialized clinical care and preventive, community-based interventions.

The Proposed 2024 HRH Reforms in Meghalaya—which encompass the ‘Proposed HRH Policy’, the new ‘Transfer and Posting Policy (2023)’, and proposed sub-cadre restructuring (HRH Update 2024)—represent a normative shift towards equity, decentralization, and systems alignment. Viewed through Walt and Gilson’s (1994) policy triangle framework, these reforms reflect changes in policy content, evolving actor constellations, and new governance processes. Yet critical equity questions remain: will creating cadres and formal structures ensure improved access for the poorest, or risk reinforcing urban concentration? Social justice perspectives, including Rawls’ distributive justice (1999) and Daniels’ accountability for reasonableness (2000), emphasize the importance of transparent processes and fair distribution of resources to underserved populations.

#### Cadres and governance: from fragmentation to differentiation

The reforms propose three new sub-cadres—Specialist, Public Health, and Teaching—to address long-standing gaps. The Specialist cadre, under a proposed Director of Hospitals and Specialized Services, seeks to retain postgraduate-trained clinicians in roles aligned with NCD management. Provisions such as pay protection, anticipatory post-creation, and controlled inter-cadre mobility emphasize career stability and motivation (Franco et al. 2002, Dussault and Franceschini 2006). The Public Health cadre aims to institutionalize preventive care, disease surveillance, and technical policy leadership critical components in addressing the social gradient of NCDs (Reddy 1993, Sheikh et al. 2014). The Teaching cadre focuses on strengthening local HRH production and reducing dependency on external training institutions, thereby supporting long-term system resilience (Frenk et al. 2010).

From a social justice perspective, this move towards functional differentiation aligns institutional design with epidemiological realities and workforce aspirations. However, equity gains will not follow cadre creation alone. Without matching reforms in resource allocation, infrastructure, and deployment governance, new cadres risk reproducing urban concentration in Shillong - the state capital and district capitals. Linking these reforms to broader equity frameworks also requires recognizing that effective NCD care depends not only on specialist availability but also on primary care integration,long-term system redesign for chronic conditions, supportive infrastructure, and the social determinants of health (WHO 2002, Epping-Jordan et al. 2004, WHO 2016). Equity-sensitive reforms must therefore be tied to broader system redesign, integrating principles of distributive justice and social determinants of health (WHO 2010, Bukhman et al. 2020).

#### Training and workforce production: from dependence to local capacity

Meghalaya’s dependence on centrally allocated training seats has constrained its ability to tailor workforce production to state-specific health priorities. Despite rising NCD needs, shortages persist in cardiology, oncology, and endocrinology, alongside a weak pipeline for public health professionals. The reforms propose partial solutions through the Teaching cadre and *ad hoc* or lateral entry into high-need specializations but do not outline expanded in-state pre-service programmes or mechanisms to influence national seat allocations.

Justice-oriented reforms would require explicit measures to expand access for tribal and rural youth, strengthen social accountability among training institutions, and ensure context-relevant training. The reforms also mandate 30 hours of continuing medical education (CME) every 5 years, a measure intended to strengthen professional development and system responsiveness. Training strategies must also align with chronic care models, equipping generalists, nurses, and primary care teams with competencies for integrated, long-term NCD care (Epping-Jordan et al. 2004, WHO 2016). Expanding pathways for rural and tribal youth thus represents not only a workforce strategy but also an equity-sensitive intervention (Bukhman et al. 2020).

#### Recruitment and entry: towards timely and need-based access

Recruitment inefficiencies marked by irregular cycles and prolonged vacancies, particularly in rural and hard-to-reach areas, have long constrained Meghalaya’s health system. The proposed reforms address this through the establishment of the Meghalaya Medical Recruitment Board and new provisions for direct specialist entry, representing significant process reforms intended to reduce bureaucratic delays and better align workforce supply with system demand.

From a systems perspective, streamlined recruitment enhances service delivery agility. From a justice perspective, it can improve access to care in historically underserved districts (WHO 2010). However, recruitment strategies must also contend with persistent structural barriers like geographic remoteness, weak transport infrastructure, and socio-economic marginalization that have historically deterred health professionals from serving tribal and rural communities (CSDH 2008, Rasanathan et al. 2011).

#### Deployment and retention: from punitive bonds to incentive-based equity

The reforms, particularly the 2023 Transfer and Posting Policy, introduce a major shift in deployment governance by categorizing facilities as Accessible, Difficult, or Inaccessible, and linking initial postings in underserved areas to career advancement. Specialist deployment is intended to be qualification-aligned and overseen by a state-level Transfer Committee and the Electronic Human Resource Management System (EHRMS) designed to enhance transparency and reduce political interference. However, their effectiveness will depend on implementation fidelity, which remains uncertain.

The proposed rural service incentive package, including compulsory rural posting (CRP) allowances, non-practicing allowance, and compensatory leave, lacks detailed financial commitments in the reviewed documents. Evidence indicates that punitive bond mechanisms have been ineffective in improving rural retention, whereas strategies such as hardship allowances, career fast-tracking, and housing support are more effective in encouraging voluntary rural service (Willis-Shattuck et al. 2008). Specialist deployment is unlikely to reduce inequities unless supported by enabling conditions such as reliable infrastructure, transport, and culturally competent care in tribal-majority and low-income areas where structural disadvantage and geographic remoteness intersect (Epping-Jordan et al. 2004, Solar and Irwin 2010).

#### Performance management and career progression: towards role clarity and accountability

The conflation of clinical and administrative roles has historically led to the loss of skilled clinicians to full-time bureaucratic positions, undermining both service quality and morale. The reforms introduce dual-track career pathways, separating clinical progression from administrative advancement. This structure is intended to retain experienced clinicians in service delivery roles while enabling those with managerial aptitude to pursue leadership positions.

Evidence suggests that linking promotions to service quality, patient outcomes, and public health indicators rather than administrative tenure strengthens both accountability and equity (Braveman and Gruskin 2003). The integration of transparent appraisal mechanisms and continuous professional development as part of these reforms could reinforce alignment between workforce performance and system objectives. Embedding equity-sensitive outcome indicators such as improved NCD coverage in rural and tribal districts, gender-sensitive career progression, and reductions in geographic disparities would ensure that performance management addresses social determinants rather than reinforcing urban or elite biases (Sen 1999, George 2007).

### Policy processes and actor dynamics related to the proposed 2024 HRH reforms: implications for health justice in Meghalaya

Analysis of policy processes and actor dynamics was guided by Walt and Gilson’s Policy Triangle, focusing on context, actors, content, and process. This lens enabled a critical examination of how the formulation and planned implementation of the reforms reflect underlying power relations and governance arrangements and their implications for equity in NCD care.

#### Policy formulation: inclusion, power, and implementation considerations

Historically, HRH policymaking in Meghalaya has been dominated by technocratic and top-down processes, with limited engagement of frontline providers, civil society, or local governance structures. Such procedural insularity often produced policies that reflected managerial logic rather than contextual realities (George et al. 2015). From a health justice perspective, such exclusion undermines fair representation and distributive equity (Daniels 2000, Rifkin 2014).

The development process of the Proposed 2024 HRH Reforms represents a notable shift. A state-level committee including government officials, professional associations (e.g. Meghalaya Medical Association), and technical partners was constituted for policy design. The public call for feedback on sub-cadre proposals indicates an emergent openness to participatory deliberation (Government of Meghalaya 2023a, 2023b). This aligns with ideals of ‘inclusive governance’ (Cornwall and Gaventa 2001), which calls for embedding the voices of those closest to health inequities in decision-making.

Despite these shifts, policy communication remains largely formalistic. The reliance on Gazette Notifications and Government Orders secures legal legitimacy but constrains accessibility for frontline workers and communities. Effective implementation will require culturally and linguistically responsive communication strategies to promote ownership and behavioural uptake (Gilson and Raphaely 2008).

#### Actor constellations and inter-sectoral dynamics

Implementation capacity depends heavily on mid-level managers, who mediate policies within contexts marked by high turnover, weak infrastructure, and political pressure. The proposal within the reforms to constitute a State Transfer Committee represents an attempt at institutional oversight, but its effectiveness in ensuring equitable deployment and resisting elite capture remains uncertain.

Beyond the health department, HRH outcomes are shaped by other sectors such as education, transport, and rural development, which influence training, infrastructure, and retention. Yet, inter-sectoral collaboration in Meghalaya remains weak, constrained by siloed mandates and fragmented governance arrangements (Rasanathan et al. 2011).

Faith-based organizations and community groups, which hold significant influence in rural service delivery, remain largely excluded from formal HRH policymaking. Their engagement through participatory co-production or accountability mechanisms could enhance responsiveness and trust (Joshi and Houtzager 2012, Olivier et al. 2015).

While external actors such as WHO, national think tanks, and development partners provide valuable technical expertise and guidance, their influence risks privileging technocratic over locally resonant solutions unless carefully mediated (Biesma et al. 2009). Professional associations and regulatory bodies (e.g. Meghalaya Medical Council) also play a double-edged role, advocating for workforce interests but potentially reinforcing corporatist logics without counterbalancing mechanisms of public accountability.

#### Power, accountability, and the political economy of the proposed human resources for health reforms

The reforms reflect a reconfiguration of power and legitimacy within the HRH policy arena. As Shiffman and Smith (2007) argue, actor power is shaped by legitimacy, access, and framing capacity. For the Proposed 2024 HRH Reforms to achieve transformative equity outcomes, structured representation of marginalized groups including rural, tribal, and gender-diverse health workers is essential.

While the reforms demonstrate normative alignment with principles of health justice, mechanisms of downward accountability remain limited. Tools such as social audits, public hearings, or citizen charters proven to enhance responsiveness in other health systems are absent from the current design. Institutionalizing such mechanisms will be critical to ensuring that policy rhetoric translates into lived equity gains (Meier and Fox 2008).

#### From reform intent to justice-oriented practice

Taken together, the Proposed 2024 HRH Reforms represent a progressive reimagining of workforce governance, with differentiated cadres, digitalized oversight, and emergent participatory forums. However, the transition from policy intent to equity outcomes requires more than institutional redesign. It necessitates redistribution of power, inter-sectoral collaboration, grassroots inclusion, and sustained transparency. Only by embedding participatory and accountable governance mechanisms can the reforms fulfil their transformative potential and respond effectively to the rising burden of NCDs with justice and integrity.

### Prospective policy analysis: impact on non-communicable diseases and health equity

To assess the potential long-term impact of proposed reforms on NCD management and health equity, a scenario-based prospective analysis was employed using the Intuitive Logic approach (Schwartz 2012, Wright et al. 2013). This structured foresight method was chosen because it facilitates systematic exploration of uncertainties and strategic assumptions in contexts where empirical prediction is constrained by limited implementation data (Buse 2008, Paina and Peters 2012). Rather than forecasting outcomes, the analysis developed a normative best-case scenario to provide a benchmark against which implementation planning can be critically assessed.

The scenario was informed by three principal inputs, namely trend data and systemic constraints identified through the document review (e.g. workforce shortages, disease burden, infrastructural gaps); determinants of success articulated in the Proposed HRH Policy, including governance quality, institutional capacity, resource allocation, and stakeholder engagement; and validation from informal consultations, which were used to refine assumptions and ensure contextual relevance. These inputs were systematically synthesized to delineate the enabling conditions, anticipated workforce distribution patterns, system-level outcomes for NCD prevention and management, and the broader equity implications of an optimal reform trajectory.

#### Scenario: best case (high success with strong equity focus)

**Assumptions.** This scenario presumes strong and sustained political will underpinned by an explicit and actionable commitment to health equity. Funding for HRH and NCD programmes is adequate, equitably allocated, and consistently available. Effective multi-sectoral collaboration, including robust partnerships with community-based organizations, is established. Monitoring and evaluation systems are well-developed, generating disaggregated data to track inequities and inform corrective action (Braveman and Gruskin 2003). Most HRH policy objectives outlined in the proposed reforms are implemented with deliberate attention to equity.

**Human resources for health outcomes.** HRH systems are significantly strengthened, enabling timely identification and redress of workforce inequities. Production, attraction, and retention of skilled HRH, including specialists in NCD-relevant fields, are enhanced through new cadre structures and retention strategies. Deliberate mechanisms ensure improved distribution of health workers to rural and underserved areas (WHO 2010). Workforce competencies in NCD prevention, management, and cultural responsiveness are expanded through targeted training and continuous professional development initiatives detailed in the reforms. Transparent and accountable regulatory frameworks govern HRH management, including recruitment (e.g. via the new Medical Recruitment Board), deployment (as per the new Transfer and Posting Policy), and career progression (Dussault and Franceschini 2006, Lehmann et al. 2008).

**Non-communicable disease and equity impact.** Access to quality NCD services improves substantially and equitably across all districts. NCD-related morbidity and premature mortality are significantly reduced, and disparities between privileged and marginalized populations begin to narrow. Overall system resilience is enhanced, reinforcing health as a public good and fundamental right (WHO 2021).

### Analysis of scenario implications

The Proposed 2024 HRH Reforms articulate a comprehensive vision for addressing systemic workforce challenges in Meghalaya, with substantial potential to improve NCD prevention and care for historically underserved communities. However, the transition from policy design to meaningful impact depends heavily on implementation capacity and political economy factors (Gilson and Raphaely 2008, Kickbusch 2015).

Achieving equitable access, a central tenet of these reforms, requires more than technical solutions. It involves operationalizing incentive structures that support rural service, not just through financial packages but also through career development, living conditions, and supportive supervision (Lehmann et al. 2008). The absence of finalized financial details for the rural incentive package leaves a critical gap in implementation planning.

Addressing chronic shortages of specialists, a key aim of the new cadre structure, will require innovative approaches such as targeted scholarships with service obligations, expansion of telemedicine networks, and strategic institutional partnerships (Dussault and Franceschini 2006, Scott and Mars, 2015). These should be embedded in a broader HRH strategy that is socially accountable and explicitly oriented towards the most disadvantaged populations.

The effectiveness of reforms ultimately depends on strong governance. Transparent recruitment and deployment processes, fair grievance redressal, and community oversight mechanisms are essential to ensure that reforms uphold social justice and equity (Brinkerhoff and Bossert 2008, 2014). Strengthening participatory models that involve civil society in planning and monitoring HRH implementation could further enhance accountability and responsiveness (George et al. 2015). Crucially, trust must be deliberately cultivated between health workers, managers, and communities, as trust is foundational for health care to function as a social institution rather than merely a technical service (Gilson 2003).

Finally, realizing the transformative potential of the best-case scenario requires that HRH reforms move beyond clinical service delivery to explicitly build capacity for health promotion, community engagement, and multi-sectoral advocacy. These are essential for tackling the commercial and cultural determinants of NCDs, not just for expanding clinical services (WHO 2021).

### Limitations of the scenario analysis

This scenario analysis is based on a prospective interpretation of the Proposed 2024 HRH Reforms. The absence of detailed implementation strategies in several components, particularly the financial scope of recruitment and retention incentives for specialist development, limits the precision of the equity impact assessment. Moreover, the analysis primarily relies on policy text and secondary literature. While this provides valuable insights into reform design and intentions, it does not capture the nuanced dynamics of power, institutional capacity, or local experiences (Erasmus and Gilson 2008). A richer understanding of these dimensions would require primary qualitative research with stakeholders, including frontline health workers and community representatives (Walt et al. 2008).

The findings highlight the dual character of the proposed reforms. They represent an ambitious attempt to reconfigure workforce governance in ways that could advance equity and strengthen system responsiveness to NCDs, yet they remain contingent upon the operationalization of institutional capacity, distributive justice in resource allocation, and mechanisms of downward accountability. This conditionality illustrates the broader challenge of translating normative policy commitments into transformative practice within health systems that are institutionally fragmented and politically contested. The discussion that follows situates these reforms within comparative debates in HPSR, engaging specifically with questions of equity, governance, and political economy in HRH reform trajectories across LMIC contexts.

## DISCUSSION

Meghalaya's evolving HRH framework, culminating in the Proposed 2024 HRH Reforms marks a critical inflection point. Situated within an epidemiological transition where NCDs now account for 56% of the state’s DALYs (IHME 2022) and entrenched workforce challenges including specialist shortages and maldistribution ( IQVIA and MHSSP, 2022a, 2022b, 2022c), these reforms represent an ambitious effort to realign institutional structures, expand competencies, and strengthen governance.

Anchored in health systems thinking (WHO 2007, de Savigny and Adam 2009), this analysis frames HRH reform not as a linear, technocratic fix, but as part of a complex adaptive system that necessitates chronic care-oriented redesign (WHO 2002). The reforms engage multiple WHO ‘building blocks’ (WHO 2007): new cadres and transfer rules for HRH management, EHRMS for information systems, and Specialist/Public Health cadres to improve responsiveness to NCD needs. In India’s historically physician-centric system (Reddy et al. 2011), such reforms must also empower other cadres and embed equity considerations for comprehensive care delivery.

This analysis draws on equity frameworks, particularly the capabilities approach advanced by Sen (1999) and Nussbaum (2000), which conceptualize health equity not merely as the reduction of disparities but as the expansion of fair opportunities for wellbeing. The unequal distribution of specialists, with over half located in East Khasi Hills, leaving some districts without any IQVIA and MHSSP (2022a), underscores the importance of equitable deployment. The best-case scenario outlined in this study envisions a policy environment where successful implementation of reforms explicitly addresses social determinants of health (Solar and Irwin 2010), through targeted resource allocation, strategic deployment (e.g. CRP, defined tenures), and capacity-building (e.g. mandatory CME) for underserved areas. Yet, without sustained political will and resourcing, outcomes may align more closely with moderate or adverse trajectories.

From a political economy perspective success will hinge on implementation rather than policy design (Brinkerhoff and Bossert 2008, Gilson 2012). Key challenges include ensuring transparent recruitment and deployment, securing equitable rural incentives, and fostering accountability between states, health workers, and communities. Without finalized financial commitments, rural retention risks repeating the failures of punitive bond mechanisms (IQVIA and MHSSP 2022c). Transformative success depends on institutional capacity (e.g. for EHRMS, objective facility categorization), stakeholder alignment (including frontline workers and communities), and political will. Conversely, weaknesses in these areas may cause stagnation, exacerbated by physician-centric hierarchies and regional preferences (e.g. East Khasi Hills) the reforms seek to address. Adaptive governance theory (Chaffin et al. 2014) highlights the need for flexible, participatory, and feedback-driven approaches to ensure reforms remain responsive to emerging challenges. At the same time, creating and protecting ‘invited’ and ‘claimed’ spaces for participation (Gaventa 2006) is crucial to embed accountability and ensure the voices of those most affected by inequities are meaningfully integrated into HRH governance.

Several strategic imperatives emerge from this analysis. Strengthening HRH data systems is central: effective deployment depends on disaggregated, interoperable systems capable of guiding real-time management (McQuide et al. 2023). Equally critical is moving beyond placeholder policies to develop comprehensive rural retention packages that combine financial and non-financial incentives, including housing, professional development, and family support. Task-shifting, particularly through the proposed Public Health cadre, offers a pathway to reduce physician-centrism by enabling nurses and community health workers to play more active roles in NCD management. At the same time, stronger community engagement in planning and monitoring could enhance accountability and ensure that reforms reflect lived realities.

Governance mechanisms, including the State Transfer Committee and grievance redressal processes, must be transparent and embedded within participatory oversight to ensure downward accountability. Finally, long-term success depends on aligning reforms with political incentives and securing stable, equitable financing (Khan 2018), without which implementation will remain fragile.

In sum, the proposed reforms present a promising, evidence-informed framework to transform Meghalaya’s health workforce architecture. These policies address long-standing deficiencies of the older service rules and respond directly to NCD and equity challenges. Their success, however, depends on confronting the political economy of reform, institutionalizing equity through tangible measures such as rural incentives and infrastructural upgrades, enabling adaptive governance, and translating systems thinking into measurable outcomes. Ultimately, Meghalaya’s HRH reforms must be understood not as an end in themselves but as a vehicle to advance health justice and redefine the state’s social contract with its most vulnerable citizens.

## Data Availability

This study is based on a qualitative document analysis of publicly available health policy documents, government regulations, and evaluation reports related to HRH reform and broader health systems planning in Meghalaya, India. All primary state-level documents were sourced from official Government of Meghalaya websites, including: https://meghealth.gov.in/documents.html; https://www.meghssp.org/document; https://mmsrb.in/MMSRB/info/history. These sites were accessed systematically between 1 March 2024 and 30 October 2024. All documents retrieved are publicly accessible and were utilized in accordance with fair use principles for academic research. The core documents analysed in this study include: **Documents from meghealth.gov.in:** The Meghalaya Health Policy, 2021 Meghalaya Mental Health and Social Care Policy, 2022 Policy Brief—Meghalaya Mental Health and Social Care Policy, 2022 Meghalaya Medical Attendance Rules, 2021 The Meghalaya Health Service Rules (1982 and 1990) **Documents from meghssp.org (Meghalaya Health Systems Strengthening Project):** Effectiveness Satisfaction Survey Report—Human Resource Health, 2023 Preliminary Report—Human Resource Health, 2022 Specialist Discussion Report—Human Resource Health, 2022 Adaptive Evaluation of State Capability Enhancement Project—Final Report, 2024 Training Needs Assessment Report, 2023 Inception Report: Capacity Building Support for Health Staff, 2023 **National and Global Data Sources (Used for Contextual Understanding and Triangulation):** Global Burden of Disease Study, 2019 National Family Health Survey (NFHS-4 and NFHS-5) World Health Organization reports on HRH and NCDs NITI Aayog Health Index Reports Rural Health Statistics (RHS) reports Most core policy documents and national/global data sets listed above are publicly accessible via official government and organizational websites. Project-specific documents, internal reports, and collated data used in the foundational situational analyses (which informed this study) may be made available upon reasonable request to the corresponding authors, subject to any applicable data sharing agreements or confidentiality policies of the Government of Meghalaya and its partners.

## Software and analysis tools

Qualitative data derived from the aforementioned sources were analysed using NVivo 14 (QSR International), a qualitative data analysis software widely utilized in health policy research. Documents were systematically imported, organized, and coded employing a combined deductive-inductive thematic analysis approach. The coding framework was deductively guided by established theoretical constructs, including the Sonderegger et al. (2021) HRH framework and Walt and Gilson’s (1994) Policy Triangle, as well as principles of political economy and health systems thinking. Inductive coding was used to capture emergent themes specific to the Meghalaya context.

Analytical rigour was enhanced using memoing, the development of framework matrices, and the construction of node hierarchies to support thematic saturation. NVivo’s features facilitated systematic comparison of themes across diverse document types (e.g. policy, regulation, evaluation reports), different actor perspectives where discernible, and varying time periods covered by the documents.

## Reflexivity Statement

The author team comprises researchers with expertise in Global Health Policy, Health Policy and Systems Research, Human Resources for Health, Health Financing, and Health Planning. We acknowledge our positionality as external technical partners and collaborators with local stakeholders and partners. This study was approached with a commitment to understanding the local context and ensuring the analysis could contribute to equitable health system strengthening. We have reflected on potential biases related to our disciplinary backgrounds and sought to integrate a social justice perspective throughout the research process.
